# Evaluation of Antimicrobial Potential and Comparison of HPLC Composition, Secondary Metabolites Count, and Antioxidant Activity of *Mentha rotundifolia* and *Mentha pulegium* Extracts

**DOI:** 10.1155/2021/9081536

**Published:** 2021-08-30

**Authors:** Nada K. Alharbi, Souheila Naghmouchi, Mayasar Al-Zaban

**Affiliations:** ^1^Biology Department, College of Science, Princess Nourah Bint Abdulrahman University, Riyadh 11671, Saudi Arabia; ^2^National Research Institute of Rural Engineering, Water, and Forestry, University of Tunis Carthage, Street of Hedi Karay BP. N 10, Ariana 2080, Tunisia

## Abstract

In the present study, the relationship between the phenolic counts, chemical composition, and biological activities of two *Mentha* species (*Mentha rotundifolia* (MR) and *Mentha pulegium* (MP)) was analyzed. The characterization of the action mode against pathogenic bacteria and the inhibition of spore germination of two fungal species using prepared methanolic extracts were studied here for the first time. The obtained data highlighted the presence of positive correlation between the secondary metabolites contents and the biological activities of the investigated extracts. In fact, HPLC analysis showed that the major components in both the extracts were eriocitrin and rosmarinic acid (25 and 20 mg/ml and 12 and 8 mg/ml in methanolic extracts of MR and MP, respectively). Moreover, the MR extract was rich in polyphenols and presents the highest antioxidant activity than MP ones. In addition, both extracts possess an antimicrobial activity against four Gram-positive and five Gram-negative bacteria and one yeast species (*Candida albicans*) and were able to inhibit the spore germination of two fungi species (*Aspergillus niger* and *Aspergillus flavus*). But, the significant activity was observed in the presence of MR methanolic extract. The effect of time on cell integrity of *E. coli* and *L. monocytogenes* determined by time-kill and bacteriolysis assays showed that the MR extract had a rapid bacteriolytic effect compared to the MP extract, and their capacities were significant against Gram-negative bacteria than positive ones. Based on the obtained data, it can be concluded that Saudi *Mentha* species have high pharmacological and industrial importance and they can be used in preparation of food or drugs.

## 1. Introduction

Several programs have been carried out to discover and develop numerous antimicrobial agents of biological, chemical, synthetic, or natural origin. Indeed, the discovery of antimicrobial drugs in the nineteenth century revolutionized the history of medicine by making it possible to treat many deadly bacterial diseases. Due to the appearance of resistant strains to the usual antibiotics, a change in the clinical spectrum of pathogens, and the emergence of pathogenicity in usually saprophytic strains in our environment, these antibiotics have suffered a decline in their microbial action potential [1, 2]. This unwanted change in the effectiveness of antibiotics and the loss of their important role as the primary agent in the treatment of infectious diseases has excited researchers to find another alternative that can play the same role with more benefit and less disadvantages [3]. In recent times, the field of modern medicine has increasingly focused interest in synthesizing drugs from plant origin. One of the important traditional sources of raw materials for medicine as well as for production of new preservatives was medicinal plants [4]. Due to their chemical composition and secondary metabolites, the plant extracts play important roles in disease resistance and inhibit the growth of toxic microorganisms in food [5–9].

Food poisoning is caused by a variety of factors, but food poisoning by microorganisms occupies a high percentage. In fact, the major food poisoning-causing bacteria are *Escherichia coli*, *Salmonella* spp, *Staphylococcus aureus*, and *Bacillus* spp [10]. Generally, there are three types of food preservatives: inorganic compounds, organic compounds, and natural food preservatives. Given that the risk and stability of inorganic and organic compounds are increasing [11], it has thus become necessary to develop natural antimicrobial agents.

The genus *Mentha*, belonging to Lamiaceae family, comprises around 25–30 species that grow in the temperate regions of Eurasia, Australia, and South Africa [12]. The most popular species of *Mentha,* in Saudi Arabia, is *Mentha pulegium* which is locally know as Al-Medina mint [13]. In addition to *M. pulegium*, *Mentha rotundifolia* L. is one of the mint species which have significant importance, both medicinally and commercially. In addition, *Mentha* spp has been used as a folk remedy for treatment of nausea, bronchitis, flatulence, anorexia, ulcerative colitis, and liver complaints due to its anti-inflammatory, carminative, antiemetic, diaphoretic, antispasmodic, analgesic, stimulant, emmenagogue, and anticatarrhal activities [14, 15]. Indeed, the different parts of *Mentha* spp are frequently used in herbal teas or as additives in commercial spice mixtures for many foods to offer aroma and flavour.

Several researchers have determined the chemical composition and biological activities of *Mentha* extract, but according to our knowledge, there are no available studies focused on the characterization of the action mode of *Mentha* species against bacteria. In addition, the biological activities, in particular, antibacterial, anticandidal, and antifungal capacities, of this plant harvested in Saudi Arabia have been scarcely studied. Therefore, the aims of the current investigation were, firstly, to determine the contents of secondary metabolites in two *Mentha* species (*M. pulegium* and *M. rotundifolia* L.) leaves and their antioxidant activity. Then, the antibacterial, anticandidal, and antifungal activities were evaluated using two methods. The characterization of the action mode against *E. coli* and *L. monocytogenes* was also assessed by time-kill and lysis assays.

## 2. Materials and Methods

### 2.1. Plant Material

The leaves of *Mentha rotundifolia* L. and *Mentha pulegium* were collected separately from an agronomical region situated in Al-Kharj in Saudi Arabia and identified according to the “Flora of the Kingdom of Saudi” [16]. The samples were dried in the shade away from light at room temperature.

### 2.2. Methanolic Extract Preparation

After drying in the dark at room temperature, the leaves of *M. rotundifolia* L. and *M. pulegium* were finely ground using blade-carbide grinding (IKA-WERK Type: A: 10). Stirring with 100 ml of pure methanol (80%) at room temperature for 24 h was carried out to extract the samples separately (10 g). Then, the concentration of the extract to dryness was done using the rotary evaporator. The prepared methanolic extracts were stored in a dark glass at 4°C until analyses.

### 2.3. Phenolic Compound Content

The Folin–Ciocalteu reagent was utilized to estimate the total phenolic concentration in the methanolic extracts obtained from two *Mentha* species. In fact, the method previously reported by Slinkard and Singleton [17] was used. The results are presented as mg gallic acid equivalents per gram dry weight (mg GAE/g DW) through the calibration curve with gallic acid.

In order to determine the total flavonoid contents in both tested extracts, the aluminum chloride colorimetric method previously described by Djeridane et al. [18] was used. The obtained data are given as rutin equivalents per gram dry weight (mg RE/g DW).

### 2.4. Chromatographic Conditions

The phenolic composition of two evaluated extracts was estimated using high-performance liquid chromatography (HPLC). In fact, the separation of the phenolic compound was realized using the Beta Basic-18 C18 column (5 *μ*m, 250 × 4.6 mm ID, Thermo Hypersil). The sample was injected at a quantity of 50 *μ*l, after filtering through a 0.45 *μ*m membrane Millipore chromatographic filter. The compounds of *M. rotundifolia* and *M. pulegium* were analyzed using an acetonitrile/water gradient with formic acid added according to the solvent program, i.e., solvent A with 1.5% formic acid in acetonitrile and solvent B with 1.5% formic acid in water, starting from 15% A up to 45% A in B by 25 min. The peaks were monitored at 280 and 330 nm. The flow rate was maintained at 1.0 ml/min. The comparison of the retention time and UV-Vis spectra of the phenolic chromatogram of the fraction with those of pure standards purchased from Sigma ((eriocitrin (45714), hesperidin (50162), narirutin (G003936); luteolin (L9283), rosmarinic acid (536954), and caffeic acid (C0625)) was used for identification of each obtained peaks

### 2.5. Antioxidant Activity

#### 2.5.1. Free Radical Scavenging Activity (DPPH)

The antioxidant activity of methanolic extracts of two *Mentha* species was firstly carried out using the 2, 2-diphenyl-1-picrylhydrazyl (DPPH) radical scavenging assay according to the published method by Hatano et al. [19]. The scavenging activity was estimated using the following equation: scavenging effect (%) = (100x (*A*_0_ − *A*_1_/*A*_0_)), where *A*_0_ and *A*_1_ are the absorbance of the control and the sample, respectively. The concentration of extract that could scavenge 50% of the DPPH radicals (IC_50_) was calculated.

#### 2.5.2. Free Radical Scavenging Ability Using ABTS Radical Cation

The ability of the investigated extracts to scavenge the ABTS radical cation was used to determine the antioxidant activity of both *Mentha* species. In fact, the method previously described by Re et al. [20] was utilized. The results are presented as the percentage of inhibition of the ABTS cation radical by the samples calculated using the following formula: scavenging effect (%) = ((*A*_0_ − *A*_1_)/*A*_0_) × 100, where *A*_0_ is the absorbance of the blank sample and *A*_1_ is the absorbance of the sample.

### 2.6. Evaluation of Antimicrobial Activity

Four Gram-positive (*Staphylococcus aureus* ATCC 6538, *Bacillus cereus* ATCC 1247, *Listeria monocytogenes* ATCC 7644, and *Enterococcus faecalis*), five Gram-negative (*Escherichia coli* ATCC 8739, *Pseudomonas aeruginosa* ATCC 9027, *Salmonella arizona* ATCC25922, *Salmonella typhimurium* NCTC 6017, and *Klebsiella pneumoniae*) pathogen bacteria, two fungi (*Aspergillus niger* and *Aspergillus flavus*), and one yeast (*Candida albicans* ATCC 2091) species were used to evaluate the antibacterial and antifungal activity of two *Mentha* species referring to two methods. Three different media were used in the current study: Trypto-Caseine Soy Agar (TSA) for growing the bacterial strains, Potato Dextrose Agar (PDA) for fungal species, and Sabouraud Dextrose Agar (SDA) for *Candida albicans*.

Preliminary screening for antimicrobial activity of *M. rotundifolia* and *M. pulegium* methanolic extracts was performed by disc diffusion assay [21, 22]. The antibiotics used as positive control for bacteria and fungi were gentamicin (10 *µ*g/disc) and amphotericin B (20 l g/disc), respectively. Negative control corresponds to the disc without sample. The disc contains the solvent that was employed to determine the solvent activity. The diameter of the growth-inhibition zone (including disc diameter of 6 mm) was used to estimate the qualitative antimicrobial activity of the tested extract.

The quantitative antimicrobial activity of investigated extracts was evaluated using the determination of minimum inhibitory concentrations (MICs) and minimum bactericidal concentrations (MBC). For that, the broth dilution method as described by Aouadhi et al. [22] was used. Microbial growth was indicated by the presence of turbidity and a “pellet” at the tube bottom. MIC was recorded visually as the lowest concentration in each row that completely inhibited bacterial growth. MBC is usually an extension from the MIC, where the microorganisms are quantitatively indicating the minimum concentration at which no viable organism appears in the culture [22].

### 2.7. Inhibition of Spore Germination of Fungal Species

The method of Athukorala et al. [23] was adopted to study the effect of both the methanolic extracts on inhibition of spore germination of two fungi species (*Aspergillus flavus* and *Aspergillus niger*). The results are expressed as the percentage of the inhibition of spore germination (*I%*) determined by microscopic examination using the following formula: *I* (%) = (*C* − *E*)/*C* × 100 (where *E* is the number of spores in the tube containing the suspension of spores + extract and *C* corresponds to the number of spores counted in the control tube).

### 2.8. Primary Mode of Action of Methanolic Extracts

The mode of action of two methanolic extracts was assessed by determining the effect of time on cell integrity using time-kill studies and bacteriolysis assay. Two microorganisms representative of Gram-negative and -positive bacteria (*E. coli* and *L. monocytogenes*, respectively) was utilized in this assay.

#### 2.8.1. Time-Kill Studies

Time-kill studies allow the characterization of the antibacterial activity of tested extracts by evaluating the reduction of bacterial count in the presence of extracts at their MIC over several hours. In fact, in the current study, the method described by Klepser et al. [24] and modified by Viljoen et al. [25] was used to evaluate the effect of *M. rotundifolia* and *M. pulegium* methanolic extracts against two representing Gram-positive and -negative bacteria. Activities of tested products, used at their MIC, were evaluated against *E. coli* and *L. monocytogenes* by measuring the reduction in the number of CFU per milliliter over 24 h. A microbial load of 10^2^ CFU was utilized as the limit of quantification by this method [26].

#### 2.8.2. Bacteriolysis

The bacteriolysis assays of tested extracts against *L. monocytogenes* and *E. coli* were assessed according to the standard method described by Carson et al. [27] and Guinoiseau et al. [26]. The results are presented as a ratio (in percent) of the OD_620_ at each time point to the OD_620_ at 0 min.

### 2.9. Statistical Analysis

The obtained data are expressed as means ± standard deviation of three replications. Tukey's post hoc tests were used to estimate the significant differences between means, and *P* values less than 0.05 were regarded as significant using SPSS 14.0 software for Windows (SPSS Inc., Chicago, IL).

## 3. Results and Discussion

### 3.1. HPLC Analysis

The chemical composition of both the methanolic extracts was determined using HPLC. The comparison with retention time and fragmentation patterns of standard compounds was used as the method for the identification of different compounds. In addition, the quantification was carried out by the external standard method from integrated peak areas of samples at 280 nm (UV absorption maximum of flavanone glycosides). As shown in Table 1, the presence of seven polyphenolic compounds can be highlighted: eriocitrin, hesperidin, narirutin, luteolin, isorhoifolin, rosmarinic acid, and caffeic acid. Despite both the extracts possessing the same chemical composition, significant variations in the concentration of the identified compounds were registered. In fact, the highest content in total polyphenols was detected in the methanolic extract of *M. rotundifolia*. The composition of *Mentha* species in polyphenols was earlier examined by Peterson and Simmonds [28], Fatiha et al. [29], and Riahi et al. [15].

The constituents observed for both extracts are well known as bioactive compounds with antioxidant, antimicrobial, anti-inflammatory, and analgesic activities. This evidence justifies the traditional and popular use of their aerial parts.

### 3.2. Secondary Metabolites Contents and Antioxidant Activity

The biological activity and the secondary metabolites of some species belonging to the *Mentha* genus were evaluated by some researchers [9, 29, 30], but there are no published data on *Mentha* species harvested in Saudi Arabia. In the current investigation, two *Mentha* species were screened for their secondary metabolites contents and biological activities.

The determination of total phenols and flavonoids contents in methanolic leaf extracts was performed by the spectrophotometric method using the Folin–Ciocalteu and the aluminum chloride reagents, respectively. The obtained data showed the existence of variation in secondary metabolites concentrations according to *Mentha* species. In fact, significant difference in the amount of total polyphenols was observed between the *M. rotundifolia* (74.45 GAE mg/g DW) and *M. pulegium* (48.4 GAE mg/g DW) extracts. However, the flavonoid contents are almost the same in both extracts (28.87 RE mg/g DW and 29.3 RE mg/g DW for *M. pulegium* and *M. rotundifolia*, respectively).

Our study confirms the results obtained in previous work which demonstrated that different *Mentha* species were rich in polyphenols. For example, the content of polyphenol in *M. pulegium* extract from Tunisia was 43.4 mg GAE/g DW [9]. In the same way, Karray-Bouraoui et al. [31] reported wide ranges varying from 20.1 to 56.6 mg GAE/g DW of polyphenols in the methanolic extract of *M. pulegium*. In addition, Fatiha et al. [29] demonstrated that the *M. spicata* methanolic extract possesses the highest phenolic content (12.0 ± 0.3 mg GAE/g) than *M. pulegium* and *M. rotundifolia* extracts and *M. rotundifolia* showed the dominant flavonoid content (3.3 ± 0.1 mg QE/g). Moreover, Nickavar et al. [30] evaluated the contents of secondary metabolites in five Iranian *Mentha* species and showed that *M. rotundifolia* presents the highest concentration of polyphenols. However, Mata et al. [32] demonstrated that *M. spicata* was rich in total phenolic compounds than *M. pulegium*.

Concerning antioxidant activity, Table 2 shows that IC_50_ values varied significantly among *Mentha* species. Based on the fact that the extract which had lower IC_50_ value indicated higher antioxidant activity, the *M. rotundifolia* methanolic extract presents the highest antioxidant activity compared to *M. pulegium*, with IC_*50*_ in the presence of ABTS and DPPH being 44 *µ*g/ml and 21 *µ*g/ml, respectively (Table 2). In addition, it can be concluded that the high contents of total phenolic compounds contributed to the essential antioxidant activity of *M. rotundifolia*. Moreover, the chemical composition of both tested *Mentha* species had an important role in their biological activities. Some investigations demonstrated that rosmarinic acid, caffeic acid, and eriocitrin can be used as an antioxidant, scavenging superoxide, and hydroxyl radicals and can inhibit oxidation of low-density lipoproteins [33]. We also noticed that both the tested methanolic extracts were rich in the same phenolic compounds.

Some researchers have found that the total phenolic content in *Mentha* species was proportional to antioxidant activity. In fact, Hajlaoui et al. [14] showed the existence of a linear correlation between antioxidant potential and phenolic content of two *Mentha* species (*M. pulegium* and *Mentha longifolia*). In order to verify this idea, correlation coefficients (*r*) between antioxidant capacities (DPPH) and phenol contents in two Saudi *Mentha* leaf extracts were determined and showed that high antioxidant activity corresponded to high total phenolic contents in the tested extracts. In fact, polyphenolic compounds seem to have an important role in stabilizing lipid oxidation and to be associated with antioxidant activity [34].

Based on the literature, it can be noticed that *M. rotundifolia* and *M. pulegium* methanolic extracts exhibited significant antioxidant activity than that described for the same species collected from other countries. For example, the IC_50_ of *M. pulegium* and *M. rotundifolia* methanolic extracts harvested in Algeria were 42.7 *µ*g/ml and 71.3 ± 2.6 *µ*g/ml, respectively [29].

### 3.3. Antibacterial Activity

In the present report, the *in vitro* antibacterial activity of methanolic extracts of two Saudi *Mentha* species against Gram-positive and Gram-negative bacteria was studied.  Table 3 shows the qualitative (determination of inhibition zones) and quantitative (determination of minimum inhibitory concentration (MIC) and minimum bactericidal concentration (MBC)) results of antibacterial activity. Indeed, the two investigated methanolic extracts presented significant antibacterial activity against all tested bacteria, but variations in the degree of this activity according to the *Mentha* and microorganism species were observed. In fact, the *M. rotundifolia* methanolic extract registered highest inhibition zone diameters with IZ ranging from 12 mm (*B. subtilis*) to 17 mm (*E. coli*). The lowest value was observed in the presence of *M. pulegium* methanolic extract for *B. subtilis*. The results of bacteriostatic and bactericidal activities of both extracts against tested bacteria are listed in Table 3 and confirmed the disc diffusion results. In fact, the tested extracts possess significant antibacterial activity with the MIC values in the range of 6.25–12.5 *µ*g/ml for *M. rotundifolia* and 12.5–25 *µ*g/ml for *M. pulegium*, respectively.

According to the obtained data, it is possible to conclude that the methanolic leaf extract of *M. rotundifolia* can be considered as significant antibacterial agents. These results are not in agreement with those obtained by Ghazghazi et al. [9] and Gulluce et al. [35] who observed that the methanol extract from aerial parts of *M. pulegium* and *M. longifolia* ssp. *longifolia* plants showed no antimicrobial activities. Recently, Riahi et al. [15] indicated that the *M. rotundifolia* methanolic extract exhibited antibacterial potential against five bacterial strains.

As for the secondary metabolites contents, same differences were found between the antimicrobial activities of methanolic extracts of two *Mentha* species. Our results suggest that the notable antibacterial property can be attributed to their phenolic compounds. Indeed, *M. rotundifolia* leaf extracts exhibited a high amount of bioactive phenolic compounds and notable antimicrobial activity than the *M. pulegium* extract. In the same way, previous findings highlighted the presence of positive correlation between total phenolic contents and antimicrobial activity [14, 36].

### 3.4. Characterization of the Action Mode of Methanolic Extracts

Generally, the target organ of active compounds in the plant extract is the cell membrane of bacteria. In fact, Hamouda and Baker [37] showed that active constituents might attack the cell wall and cell membrane, thereby destroying their permeability barrier and causing the release of intracellular constituents like ribose and sodium glutamate. Also, they interfere with electron transport, nutrient uptake, protein and nucleic acid synthesis, and enzyme activity, leading to the inhibition of bacterial growth. In the current finding, the characterization of the mechanisms of action of two *Mentha* species methanolic extracts was carried out for the first time. In fact, experiments on cell death and bacteriolysis of evaluated extracts against two bacterial species (*E. coli* and *L. monocytogenes*) were used to measure the effects induced by time-dependent treatments on cell viability.

#### 3.4.1. Dynamics Action of the Methanolic Extract: Time-Kill Analysis

**Figure 1:**
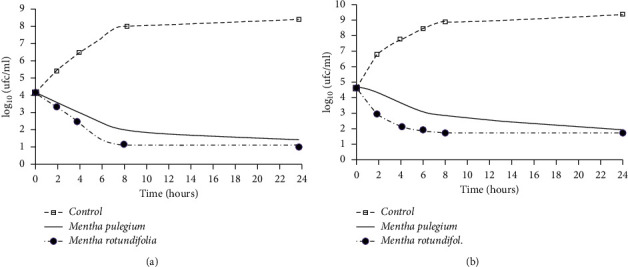
Time-kill curves *E. coli* (a) and *L. monocytogenes* (b) cultures untreated and treated with the methanolic extract of two *Mentha species* at a concentration corresponding to the MIC.

In order to study the antibacterial action mode of evaluated extracts, the growth of two selected bacteria (*L. monocytogenes* and *E. coli*) were monitored, in the absence and presence of extracts at a concentration corresponding to the MIC over a period of 24 hours. As presented in Figure 1, the control population shows a classic growth curve with three phases (growth, stationary, and decline phases). In the presence of each extract at a concentration corresponding to MIC, the shape of the growth curve is reversed and the three phases no longer appear indicating the cessation of growth of both bacterial strains after incubation for 24 hours at 37°C. In fact, after 2 hours of treatment, the number of viable cells decreased. It reaches the limit of detection (inhibition of about 50% of the initial population = 2log (CFU/ml)) after 8 h and 4 h for *E. coli* and *L. monocytogenes,* respectively, in the presence of *M. rotundifolia* and after 8 h for both bacterial species in the presence of *M. pulegium* methanolic extract. Based on these results, it can be signaled that the bactericidal effect of *Mentha* methanolic extracts depends on the duration of incubation, bacterial species, and evaluated *Mentha* species. In fact, *M. rotundifolia* had the highest bactericidal activity than *M. pulegium* and shows more efficiency against *E. coli* (Gram-negative bacteria) than *L. monocytogenes* (Gram-positive bacteria).

#### 3.4.2. Determination of the Lytic Action of the Methanolic Extract

The measure of the absorbance of the bacterial strains in the absence and presence of each extract, at a concentration corresponding to MIC, was used to determine the lytic action of the methanolic extract of two Saudi *Mentha* species against two bacterial species (*L. monocytogenes* and *E. coli*). The loss of absorbance after 2 hours of incubation is evaluated based on the initial absorbance. The results are therefore expressed as the ratio of the absorbance measured at time *T* to the absorbance at 620 nm measured at time zero ((OD_620_ (*T*)/OD_620_ (*T*_0_)) × 100).

**Figure 2:**
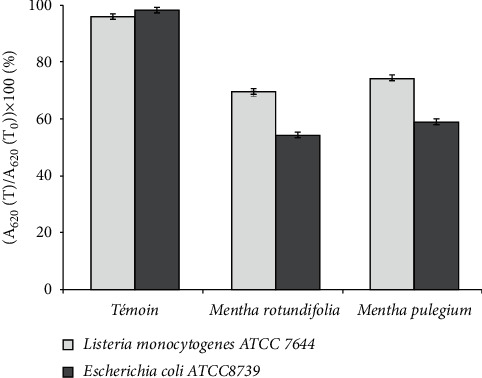
Cell integrity of *E. coli* and *L. monocytogenes* after treatment with two *Mentha* extracts at a concentration corresponding to the MIC.

Figure 2 shows that in the case of control (without extract), the absorbance of two bacterial strains are around 100% indicating the absence of cell lysis. However, the addition of extract caused a decrease in the initial absorbance of both bacteria. Indeed, the optical density decreased to 50% and 70% for *E. coli* and *L. monocytogenes*, respectively, in the presence of both *Mentha* species.

Usually, some antimicrobial agents destroy the bacterial membrane irreversibly leading to cell death by a lytic process [37–39]. Indeed, the obtained data confirmed the time-kill assay in that the methanolic extract of two *Mentha* species had a bacteriolytic effect against two different categories of bacteria, Gram-positive and Gram-negative, which are not of the same potency. Indeed, *E. coli* was more sensitive to the effect of the tested extract than *L. monocytogenes*. These results are in conformity with those of Horne et al. [40]. In fact, these authors demonstrated that the essential oils of oregano, rosewood, and thyme generate lytic effects against *Streptococcus pneumoniae*. However, in other works, we observed that plant extracts compromise the structural integrity of the plasma membrane and induce loss of the cytoplasm material but do not lyse bacterial cells [27, 41].

Previous works signaled that the antimicrobial effect of plant extracts was greater on Gram-positive bacteria than on negative ones. This is due to the difference in their cell wall (membrane proteins of Gram-negative organisms acting as a barrier to many environmental substances, including antimicrobial agents) [42]. In our finding, Saudi *Mentha* species had *in vitro* selective antibacterial activity on the basis of the cell-wall differences of bacterial microorganisms with the Gram-negative bacteria being more sensitive than Gram-positive bacteria.

### 3.5. Antifungal Activity and Inhibition of Spore Germination

The chemical composition and antibacterial activity of *M. rotundifolia* and *M. pulegium* methanolic extracts have been investigated by some researchers, but their antifungal and anticandidal activities have been scarcely studied. In the current study, the antifungal and anticandidal activities of two Saudi *Mentha* methanolic extracts as well as the inhibition of spore germination of *A. flavus* and *A. niger* were investigated. Generally, the main pathogenic fungal species for humans, animals, and plants are *Aspergillus niger* and *Aspergillus flavus*. In fact, they able to cause aspergillosis in immunocompromised individuals as well as postharvest disease in cereal grains and legumes [43, 44]. The antifungal activity results showed that the methanolic extracts exhibited a moderate activity against yeast (*C. albicans*) and two fungi species (*A. flavus* and *A. niger*) with the maximum inhibition zones and MIC values ranging from 10 to 13 mm and 12.5 to 25 *µ*g/ml, respectively (Table 3).

Concerning the inhibition of spore germination, the *M. rotundifolia* methanol extract exhibited the highest activity against the two fungal species with the inhibition rate around 35%, whereas with the *M. pulegium* extract, it was 50%. These data clearly showed that the Saudi *Mentha* species were able to inhibit the spore germination of *Aspergillus* spp.

## 4. Conclusion

The obtained data highlight the importance of Saudi *Mentha* species as a promising source of natural antioxidant, antifungal, and antibacterial agents for food preservation and the prevention against oxidative stress-related disease and pathogenic bacteria.

## Figures and Tables

**Table 1:** Contents of *Mentha rotundifolia* and *Mentha pulegium* polyphenolic compounds determined by HPLC.

Identified compounds	Content of compounds (mg/g of dry weight)		Retention time (min)
	*M. rotundifolia*	*M. pulegium*	
Eriocitrin	25	20.1	12.5
Hesperidin	0.75	0.5	17.5
Narirutin	0.10	0.09	16.25
Luteolin	0.22	0.2	15.5
Isorhoifolin	2.5	1.2	14
Rosmarinic acid	12	8	18.5
Caffeic acid	8.55	0.3	10.5

**Table 2:** Antioxidant activities of methanolic extracts of two *Mentha* species.

Species	DPPH (IC_50_, *μ*g/ml)	ABTS (IC_50_, *μ*g/ml)
*M. pulegium*	27.31 ± 0.94	57.08 ± 0.85
*M. rotundifolia*	21.08 ± 0.83	44.01 ± 0.96
Butylated hydroxytoluene (BHT)	20	46

Values are given as mean ± SD (*n* = 3).

**Table 3:** Antibacterial and antifungal activities of *Mentha* methanolic extracts against nine bacteria evaluated by disc diffusion and determination of MIC and MBC tests.

Strains^a^	Inhibition zone diameters (mm)^b^			MIC (*µ*g/ml)		MBC (*µ*g/ml)	
	MR	MP	Antibiotics	MR	MP	MR	MP
*Gram-negative bacteria*							
*E. coli* ATCC 8739	17 ± 0.5	16 ± 0.5	24	12.5	25	12.5	25
*S. typhimurium* NCTC 6017	15 ± 1	14 ± 0.5	23	12.5	25	25	50
*S. arizona* ATCC25922	15 ± 0.7	14 ± 0.4	23	12.5	25	25	50
*P. aeruginosa* ATCC 27853	17 ± 0.8	16 ± 0.3	21	12.5	25	12.5	25
*Klebsiella pneumoniae*	15 ± 0.5	14 ± 0.4	20	6.25	12.5	12.5	25

*Gram-positive bacteria*							
*L. monocytogenes* ATCC 7644	13 ± 0.5	12 ± 0.5	18	12.5	25	25	50
*B. cereus* ATCC1247	12 ± 0.5	11 ± 0.6	21	12.5	25	25	50
*E. faecalis*	14 ± 0.6	13 ± 0.3	19	12.5	25	12.5	25
*S. aureus*	15 ± 0.4	14 ± 0.2	20	12.5	25	25	50

*Fungus species*							
*Aspergillus flavus*	14 ± 0.4	13 ± 0.4	11	12.5	12.5	25	25
*Aspergillus niger*	16 ± 0.5	15 ± 0.3	11	12.5	12.5	25	25
*Candida albicans*	11 ± 0.6	11 ± 0.4	17	12.5	12.5	25	25

^a^The final bacterial density was around 10^5^ cfu/mL. ^b^Inhibition zone diameters (mm) produced around the wells were determined by adding 15 *µ*L of methanol extracts. MIC: minimum inhibitory concentration (*µ*g/ml); MBC: minimum bactericidal concentration (*µ*g/ml). Values are means of three measurements; ±: standard deviation.

## Data Availability

No data were used to support this study.
