# Corrigendum to “Evaluation of Antimicrobial Potential and Comparison of HPLC Composition, Secondary Metabolites Count, and Antioxidant Activity of *Mentha rotundifolia* and *Mentha pulegium* Extracts”

**DOI:** 10.1155/2022/9767418

**Published:** 2022-08-13

**Authors:** Nada K. Alharbi, Souheila Naghmouchi, Mayasar Al-Zaban

**Affiliations:** ^1^Department of Biology, College of Science, Princess Nourah bint Abdulrahman University, P.O. Box 84428, Riyadh 11671, Saudi Arabia; ^2^National Research Institute of Rural Engineering, Water and Forestry, University of Tunis Carthage, Street of Hedi Karay BP. N 10, Ariana 2080, Tunisia

In the article titled “Evaluation of Antimicrobial Potential and Comparison of HPLC Composition, Secondary Metabolites Count, and Antioxidant Activity of *Mentha rotundifolia* and *Mentha pulegium* Extracts” [1], there is an error in the first affiliation. The correct affiliation is “Department of Biology, College of Science, Princess Nourah bint Abdulrahman University, P.O. Box 84428, Riyadh 11671, Saudi Arabia” and it is corrected above.

## References

[1] Alharbi N. K., Naghmouchi S., Al-Zaban M. (2021). Evaluation of antimicrobial potential and comparison of HPLC composition, secondary metabolites count, and antioxidant activity of *Mentha rotundifolia* and *Mentha pulegium* extracts. *Evidence-based Complementary and Alternative Medicine*.

